# Eustachian tube dysfunction in OSMF- often present seldom discovered

**DOI:** 10.4317/jced.51593

**Published:** 2014-10-01

**Authors:** Sana-Noor Siddiqui, Nisheeth Saawarn, Preeti P. Nair, Pooja Singh, Harshkant P. Gharote, Karthik Hegde

**Affiliations:** 1 PG student. Department of Oral Medicine and Radiology. People’s College of Dental Sciences and Research Centre. Bhopal, India; 2 MDS. Department of Oral Medicine and Radiology. People’s College of Dental Sciences and Research Centre. Bhopal, India

## Abstract

Objectives: To evaluate the effect of OSMF on the eustachian tube function and to correlate it with various grades of the disease. Study Design: Twenty OSMF patients (40 ears) and 20 healthy controls (40 ears) were evaluated for eustachian tube function by eustachian tube function test, tympanometry and audiometry. Results: The audiometric and tympanometric analysis showed no significant differences in hearing abilities of OSMF patients and controls and between various grades of OSMF, indicating no hearing impairment. However, eustachian tube function test revealed a statistically significant difference in eustachian tube function in OSMF patients and controls. Further, there was a significant increase in severity of dysfunction with increase in severity of disease. Conclusions: From the present study it is evident that the subjective function of Eustachian tube may be affected by disease process. But, probably the amount of deviation found in function of the eustachian tube is non contributing to cause a conductive hearing loss.

** Key words:**Oral submucous fibrosis, eustachian tube function, tympanometry.

## Introduction

Oral submucous fibrosis [OSMF] is a chronic insidious disease, affecting any part of the oral cavity and sometimes the pharynx. Although occasionally preceded by and/or associated with vesicle formation, it is always associated with a juxta-epithelial inflammatory reaction followed by fibroelastic changes of the lamina propria, with epithelial atrophy leading to stiffness of the oral mucosa and causing trismus and inability to eat (1).

A variety of etiological factors including capsaicin, betel nut alkaloids, hypersensitivity, autoimmunity, genetic predisposition and chronic iron and vitamin B-complex deficiency have been suggested by various authors, the most common of which is chewing areca nut (2).

Pathogenesis of the disease is best explained by reduced phagocytosis of collagen by fibroblasts, up or down regulation of key enzymes such as lysyl oxidase, matrix metalloproteinases and tissue inhibitors of matrix metalloproteinases. The process may also be influenced by increased secretion of inflammatory cytokines, growth factors and decreased production of anti-fibrotic cytokines (2).

Symptoms include burning sensation of the oral mucosa, ulceration and pain, reduced movement and depapillation of tongue, blanching and leathery texture of oral mucosa, loss of pigmentation of oral mucosa, and progressive reduction of mouth opening (3). Advanced cases show signs of loss of hearing due to blockage of eustachian tubes and difficulty swallowing because of esophageal fibrosis (4).

Histological changes include appearance of excessive collagen fibres, constricted blood vessels, edema and excessive deposition of fibroblast and infiltration of inflammatory cells (5). In severe cases along with mucosa and sub mucosa, degenerative changes have been reported in deeper tissues as well including muscle fibres (6,7). This have been reported by Gupta SC et al. in their study where palatal and paratubal muscles showed degenerative changes in the form of atrophy, loss of cross striations and edema of myoepithelium (8). Changes in these muscles which are attached to eustachian tube and soft palate may lead to eustachian tube dysfunction and hearing impairment (9).

Gupta SC *et al.* (9) and Shah M *et al.* (10) in their studies clinically evaluated the eustachian tube function by audiometry and tympanometry and found its significant impairment in OSMF patients as compared to healthy controls. These studies substantiate both clinically and histologically that eustachian tube function and hearing ability is impaired in OSMF.

However there are no studies available correlating the eustachian tube dysfunction with various clinical stages of OSMF and its association with increase or decrease in the severity of the disease process. Therefore, this study was designed to evaluate eustachian tube function in OSMF patients and to correlate it with various clinical stages of the disease which may be helpful in assessing the morbidity and in identifying the overall prognosis to find more appropriate therapeutic interventions.

## Material and Methods

This prospective study was conducted at People’s College of Dental Sciences and Research Centre, Bhopal in India between December 2012 to August 2013 on twenty otherwise systemically healthy OSMF patients with no other oral lesions and twenty age and sex matched healthy controls with no oral mucosal lesion or deleterious habits after obtaining ethical clearance from the Institutional ethical committee.

After the clinical examination, the diagnosis of OSMF was made on the basis of natural history and characteristic clinical features of the disease which included burning sensation in mouth, intolerance to spicy food, blanching and loss of suppleness of oral mucosa, presence of palpable fibrous bands and decreased mouth opening. Since the clinical features are highly characteristic and almost pathognomic, biopsy and histopathological confirmation is not always necessary. Further, there is always a probability that the biopsy may act as an irritant in a form of mechanical trauma and the biopsy site may heal with scar formation due to decreased vascularity and impaired collagen synthesis in the mucosa, which may worsen the disease condition by further limiting the mouth opening. (11). The patients were divided into three groups on the basis of interincisal mouth opening [0-10mm, 11-20mm, 21-30mm]. Mouth opening was calculated using a metallic caliper and a scale taking incisal edges of maxillary and mandibular central incisors as reference point. Patients with inadequate reference point for measuring intericisal distance were excluded from the study.

After obtaining an informed written consent from patients they were subjected to otolaryngological examination by a qualified otolaryngologist to rule out any other disorder affecting the hearing ability and eustachian tube function.

Followed by which pure tone audiometry [PTA] was performed in a sound proof room using ALPF AD 2100 equipment. Both the ears of the subjects from study [40 ears] and control group [40 ears] were subjected to this analysis. Air conduction [AC] and bone conduction [BC] thresholds were measured for tones of 125,250,500,1000,2000 and 4000 Hz. The amount of intensity that has to be raised above the normal level is the measure of degree of the hearing impairment at that frequency. Depending upon the AC-BC Gap values, hearing impairment was quantitatively graded into several categories as follows: 0-25 dB Normal hearing, 26-40 dB Mild deafness, 41-55 dB Moderate deafness.

Tympanometric analysis was done using Interacoustic AT 235 equipment. A small probe was inserted which emits a sound of low frequency [226Hz] via a tube into the auditory canal and a continuous change of positive and negative pressure was created by the pump of the instrument in the external auditory canal in front of the tympanic membrane. The compliance [which is inversely proportional to the impedance] was measured simultaneously and shown in a graph known as tympanogram. Out of the graphs obtained Type A is considered as normal and Type B & C are considered as abnormal tympanograms.

The principle of ETF test is same as that of tympanometry. However, in ETF test multiple tympanograms are obtained for different middle ear pressures [i.e. at normal, negative and positive pressures] in three different conditions, viz. the middle ear pressure at the start of the test, after patient swallows with nose and mouth closed and finally after performing valsalva. The pressure values at maximum compliance are recorded from all the tympanograms and the shift in the compliance peaks at normal, negative and positive pressures are calculated. Shift in compliance peaks showed that eustachian tube functions were good and compliance peaks with no shift showed poorly functioning of eustachian tube.

The data so obtained was than tabulated and analysed statistically using Chi square and Spearman correlation.

## Results

Subject group comprised of 16 males and 4 females aged between 18-56 yrs [mean age being 28] and control group comprised of 15 males and 5 females aged between 21-47 yrs [mean age being 27] ( Table 1).

**Table 1:**
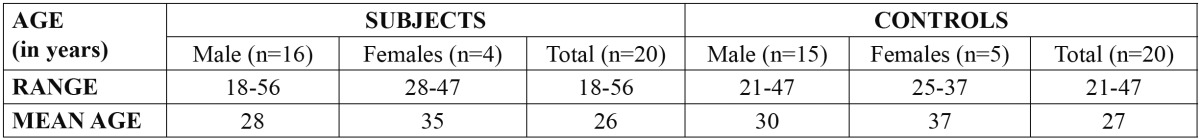
Age and sex distribution of OSMF subjects and controls:

On the basis of mouth opening OSMF patients were then divided into three groups as 0-10mm, 11-20 mm and 21-30 mm.

**Figure 1:**
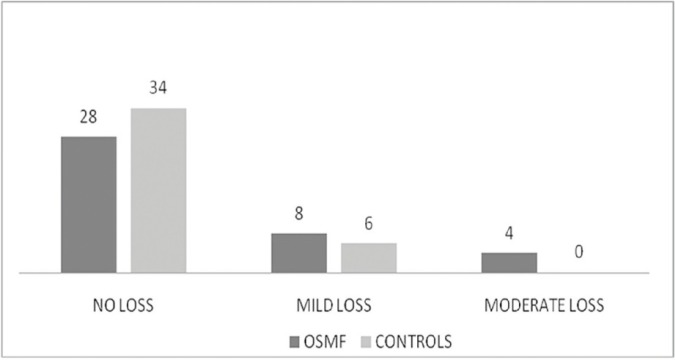
Results of Audiometry.

On PTA hearing among OSMF patients was found to be normal in 28 [70%] ears, mild loss was found in 8 [20%] ears and moderate loss was present in 4 [10%] ears. In control group hearing was found to be normal in 34 [85 %] ears and mild loss was found in 6 [15%] ears and none showed moderate hearing loss. No significant difference [p=0.088] was found on further comparison between both groups (Fig. 1).

On the further comparison between mouth opening and hearing loss on basis of pure tone audiometry no statistically significant relationship was found ( Table 2).

**Table 2:**
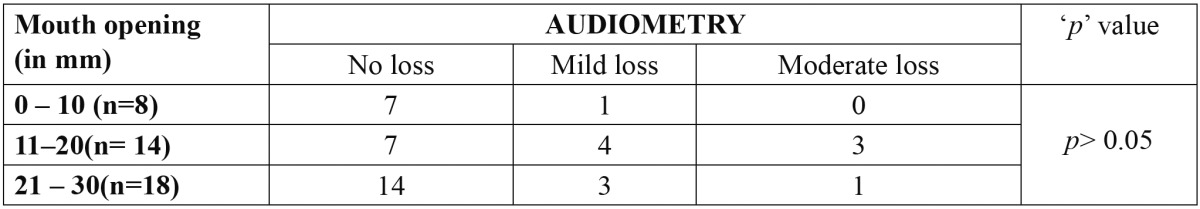
Comparison of mouth opening and hearing loss.

**Figure 2:**
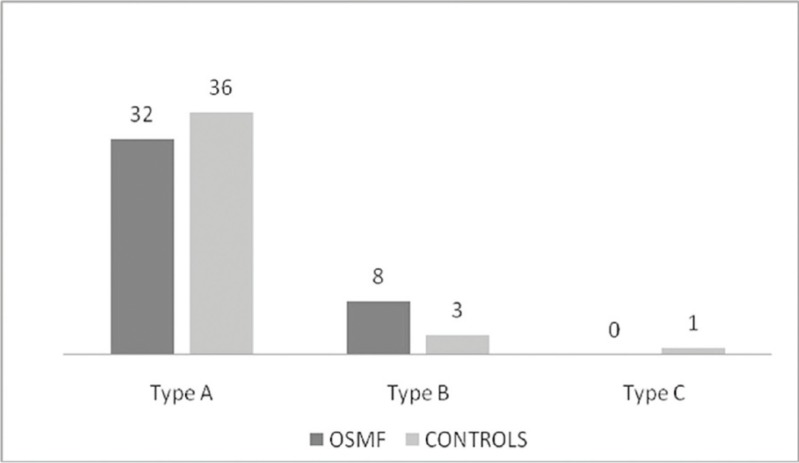
Results of Tympanometry.

On tympanometry out of 40 ears in OSMF group normal tympanogram type A curve was recorded in 32 [80%] ears while abnormal type B and type C tympanograms were recorded in 8 [20%] ears and in none respectively. In control group out of 40 ears normal tympanogram type A curve was recorded in 36 [90%] ears abnormal type B and type C tympanograms were found in 3 [7%] ears and 1 [3%] ear respectively. On comparing both groups the results of tympanometry were statistically insignificant [*p*>0.05], (Fig. 2).

On the further comparison between mouth opening and type of curves on basis of tympanometry results obtained showed no statistical significant relationship ( Table 3).

**Table 3:**
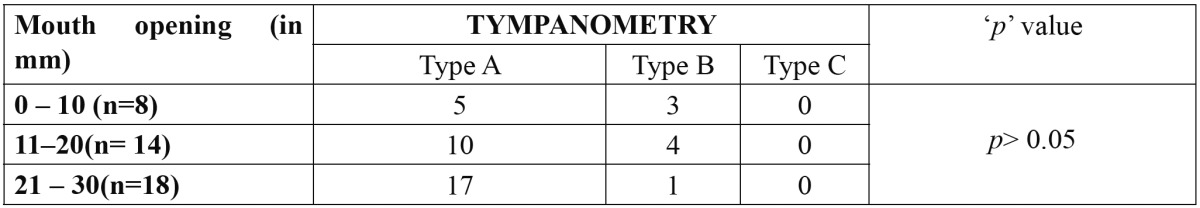
Comparison of mouth opening and types of curve.

**Figure 3:**
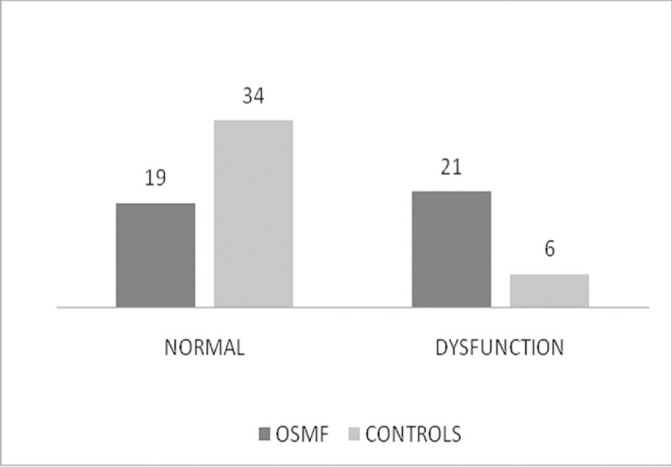
Results of Eustachian Tube Function Test.

In OSMF group out of 40 ears eustachian tube function test [ETFT] revealed no shift in compliance peaks in 21 [53%] ears and in control group no shift in compliance peaks in 6 [15%] ears with −200 daPa pressure changes after swallowing. On comparing both groups the results of ETFT were highly statistically significant [*p*=0.000], (Fig. 3).

On comparing mouth opening and eustachian tube dysfunction on basis of ETFT the results obtained were statistically significant ( Table 4).

**Table 4:**
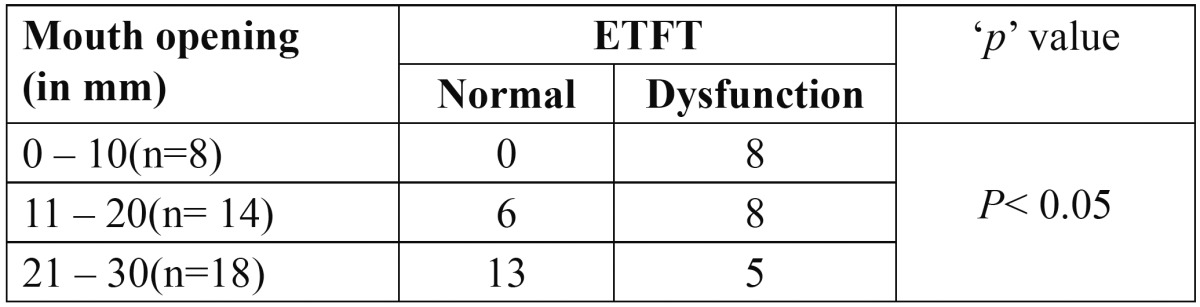
Comparison of mouth opening and eustachian tube function.

## Discussion

OSMF is a chronic, insidious, disabling disease involving oral mucosa, the oropharynx and rarely larynx (12). The disease is characterized by blanching and stiffness of the oral mucosa, trismus, burning sensation in the mouth, hypomobility of the soft palate and tongue, loss of gustatory sensation, and occasionally, mild hearing loss due to blockage of eustachian tube (13). It affects about 0.2-1.2% of the Indian population (14,15). The disease shows a male predominance (16) however few authors have reported a female predominance (17). In the present study we observed a male predominance (3:1) and the subjects ranged in the age of 18-56 years. Most of these patients were in the second and third decade of life with 15 [75%] patients being in the age group of 11-30 years. This is in accordance with the studies by Gupta *et al.* (8) and Shah *et al.* (10).

The various suggested etiological factors include capsaicin, betal nut alkaloids, hypersensitivity, autoimmunity, genetic predisposition and malnutrition (2). In the present study most of the patients were betal nut chewers.

The malignant transformation rate of oral submucous fibrosis has been found to be 4 to 13% worldwide and 7.6% in Indian population (17,18).

As the disease gradually progresses in severity finally converting into malignancy in few cases, several staging and grading systems have been proposed by various researchers based on clinical and /or (15,19) histological (15,20) features.

The clinical staging is primarily done on the basis of features like burning sensation, blanching of mucosa, vesicle & ulcer formation, (21,22) presence and extension of fibrous bands, mouth opening limitation, (21,22) restriction of tongue movement, depapillation of tongue and cheek flexibilit (21). Few authors have used the concurrent presence of other premalignant lesions and malignancy in the grading and staging of the disease (16,22).

Histological features seen in early cases of OSMF are fine fibrils of collagen, edema, hypertrophic fibroblast, dilated and congested blood vessels and an infiltration of neutrophilic and eosinophilic granulocytes, which is followed by a down regulation of fibroblast, epithelial atrophy and loss of rete pegs and appearance of hyalinization with an infiltration of inflammatory cells in later stages (5).

These pathological changes not only affect the mucosa and sub mucosa, but also the underlying muscles and deeper tissues (6). Binnie and Cawson have reported a homogenous collagenous subepithelial zone along with degeneration of muscle fibers (23). Oliver AJ *et al.* reported the presence of dense collagen bundles that were randomly oriented and extended into the underlying striated muscles (24). El Labban NG and Caniff JP in their study reported severe degenerative changes in high proportion of underlying submucosal muscle fibers exhibiting complete loss of plasma membrane with surrounding edematous fluid. (7).

Rajendran *et al.* upon electron microscopic evaluation reported focal lysis and hypercontraction of myofibres and extensive fatty infiltration between muscle bundles in biopsy specimens taken from buccal mucosa of OSMF patients (6). Gupta SC *et al.* who have taken biopsy specimens from soft palate reported degenerative changes in palatal and paratubal muscles in the form of atrophy, loss of cross striations and edema of myoepithelium (8). Palatal and paratubal muscles [levator veli palatini, tensor veli palatine, tensor tympani and salphingopharyngeous], which regulate the patency and function of pharyngeal orifice, hence may get affected, resulting in impairment of function and patency of eustachian tube. This leads to pain in the ear along with mild to moderate conductive loss of hearing (4).

As the objective of our study was to correlate the degree of hearing loss if any with the degree of mouth opening restriction, we did not use any of the proposed staging system of OSMF. Instead the patients were grouped in various groups based on their mouth opening. A difference of 10 mm in mouth opening was used for this. The patients were grouped accordingly as having mouth openings in the range of 0-10 mm, 11-20mm, 21-30 mm and 6 than 30 mm. Histological features too were not included for grading of patients as the biopsy was not done in order to avoid any mechanical trauma which can further worsen the condition by decreasing the mouth opening due to scar formation in already compromised mucosa.

On pure tone audiometry 28 [70%] ears in study group found to have normal hearing, which was consistent with 79.2% and 66.7 % ears in studies by Gupta *et al.* (8) and Shah *et al.* (10) respectively. But it was statistically insignificant [*p* =0.088] when compared with 34 [85%] ears in control group.

Abnormal tympanograms were present 20% (8) of the ears in cases of OSMF. Similar to the results of Gupta SC *et al.* [24.6 %] (8) and Shah M *et al.* [22.2%] (10). But, it was statistically insignificant [*p* >0.05] when compared with 4 [10%] normal ears in control group.

ETFT revealed eustachian tube dysfunction in 21 [53%] ears in cases of OSMF.Which was higher than the results of Gupta SC *et al.* 26.4% (8) and Shah M *et al.* 27.8% (10). Which was statistically significant [*p* =0.000] when compared with 6 [15%] ears in control group.

There were no significant differences statistically in pure tone audiometry and tympanometry results between study and control groups, but the results of eustachian tube function tests were altered, which were statistically significant when compared to control group.

From the present study it is evident that the subjective function of Eustachian tube may be affected by disease process. But, probably the amount of deviation found in function of the eustachian tube is non contributing to cause a conductive hearing loss. However, the results can be further justified by studies involving voluminous sample size and patient with more severe OSMF.
